# Combined DECS Analysis and Next-Generation Sequencing Enable Efficient Detection of Novel Plant RNA Viruses

**DOI:** 10.3390/v8030070

**Published:** 2016-03-07

**Authors:** Hironobu Yanagisawa, Reiko Tomita, Koji Katsu, Takuya Uehara, Go Atsumi, Chika Tateda, Kappei Kobayashi, Ken-Taro Sekine

**Affiliations:** 1 NARO Agricultural Research Center, 3-1-1 Kannondai, Tsukuba, Ibaraki 305-0856, Japan; yana1208@affrc.go.jp; 2 Yokohama Plant Protection Station, Ministry of Agriculture, Forestry and Fisheries, 1-7 Nagamine, Tsukuba, Ibaraki 305-0052, Japan; katsuk@pps.maff.go.jp (K.K.); ueharat@pps.maff.go.jp (T.U.); 3 Iwate Biotechnology Research Center, 22-174-4 Narita, Kitakami, Iwate 024-0003, Japan; r-tomita@ibrc.or.jp (R.T.); go-atsumi@aist.go.jp (G.A.); c-tateda@ibrc.or.jp (C.T.); 4 National Institute of Advanced Industrial Science and Technology, 2-17-2-1 Tsukisamuhigashi, Toyohira-ku, Sapporo, Hokkaido 062-8517, Japan; 5 Laboratory of Plant Molecular Biology and Virology, Faculty of Agriculture, Ehime University, 3-5-7, Tarumi, Matsuyama, Ehime 790-8566, Japan; kappei@ehime-u.ac.jp; 6 Laboratory of Plant Pathology, Faculty of Agriculture, University of the Ryukyus, 1 Senbaru, Nishihara-cho, Okinawa 903-0213, Japan

**Keywords:** deep-sequencing, dsRNA, dsRNA-binding protein, detection, blueberry, *Sobemovirus*, *Blueberry shoestring virus*, J0101

## Abstract

The presence of high molecular weight double-stranded RNA (dsRNA) within plant cells is an indicator of infection with RNA viruses as these possess genomic or replicative dsRNA. DECS (dsRNA isolation, exhaustive amplification, cloning, and sequencing) analysis has been shown to be capable of detecting unknown viruses. We postulated that a combination of DECS analysis and next-generation sequencing (NGS) would improve detection efficiency and usability of the technique. Here, we describe a model case in which we efficiently detected the presumed genome sequence of *Blueberry shoestring virus* (BSSV), a member of the genus *Sobemovirus*, which has not so far been reported. dsRNAs were isolated from BSSV-infected blueberry plants using the dsRNA-binding protein, reverse-transcribed, amplified, and sequenced using NGS. A contig of 4,020 nucleotides (nt) that shared similarities with sequences from other *Sobemovirus* species was obtained as a candidate of the BSSV genomic sequence. Reverse transcription (RT)-PCR primer sets based on sequences from this contig enabled the detection of BSSV in all BSSV-infected plants tested but not in healthy controls. A recombinant protein encoded by the putative coat protein gene was bound by the BSSV-antibody, indicating that the candidate sequence was that of BSSV itself. Our results suggest that a combination of DECS analysis and NGS, designated here as “DECS-C,” is a powerful method for detecting novel plant viruses.

## 1. Introduction

When plants exhibiting virus-like symptoms are found on a farm or during a routine plant inspection, the rapid identification of the causal agents is important for taking adequate and timely measures for their control. Conventionally, serodiagnostic tests and/or PCR assays are widely used to detect plant viruses. These methods are effective for detection of viruses commonly known to infect particular plant species. However, it is difficult and time-consuming to detect pathogenic viruses using these methods for new virus strains or infection of new host plants; in these circumstances, different assays need to be performed. For example, the pathogenic causal agents of many graft-transmissible diseases affecting woody crops remain to be elucidated; this is largely because of the difficulty in detecting and isolating candidate viruses from woody crops, and of artificially infecting new host plants to reproduce the disease symptoms. The majority of identified plant viruses are RNA viruses, which possess double-stranded RNA (dsRNA) as the genome or as a replicative form. Therefore, isolating dsRNA is an effective approach for detection of viruses. To date, CF-11 cellulose powder chromatography, digestion with adequate nuclease and/or lithium chloride precipitation have been used to isolate dsRNAs [1,2,3]. The more recently developed DECS (dsRNA isolation, exhaustive amplification, cloning and sequencing) protocol, established by Kobayashi and colleagues, provides another convenient method to isolate dsRNA and to determine sequences derived from viruses [4,5]. In this method, dsRNAs are isolated using a recombinant dsRNA-binding protein DRB4 from *Arabidopsis thaliana*, converted to complementary DNAs (cDNAs), amplified, cloned into plasmid vectors, and randomly selected clones are sequenced. These sequences can be searched against a database (e.g., Basic Local Alignment Search Tool (BLAST) search) to determine the species of the virus. The DECS method can also be used to detect RNA viruses in a small volume of plant tissues and to collect dsRNAs within a short time [5]. DECS analyses have contributed to the identification of novel viruses in Japan; for example, gentian Kobu-sho-associated virus, gentian ovary ring-spot virus, and lisianthus necrotic ringspot virus were reported for the first time using this approach [6,7,8].

DECS analysis involves a cloning step to determine the sequences of dsRNAs. When a large amount of trapped viral dsRNA is obtained, even a small number of sequencing operations (e.g., in about 16 clones) may suffice as a detection procedure because the clones are likely to contain some viral sequences. However, if a small amount of trapped dsRNA is available, then sequencing of a larger number of clones (e.g., more than 100 clones) might be needed to ensure the detection of viral sequences. The amount of viral dsRNA that accumulates in plant cells is influenced by the host plant species, the time of sampling, and the plant tissue types sampled. Furthermore, it takes considerable time to connect fragments and/or to perform rapid amplification of cDNA ends (RACE) to determine the whole genomic sequence of the virus. For this latter purpose, it is necessary to increase the number of clones that are sequenced, which makes the procedure more laborious and time consuming. Therefore, the use of a more efficient sequencing technology would significantly improve the detection sensitivity of DECS analysis and speed up the determination of whole genome sequences. One option would be to use next-generation sequencing NGS, which allows the generation of considerably larger amount of sequence data in a given time. In recent years, many new plant viruses have been detected from diseased plants using NGS technology [9,10,11,12,13,14,15]. dsRNAs have been used as the template for NGS to successfully detect plant viruses and viroids [11,15]. The technical difficulty of the low amount of viral dsRNA in the plant tissues might be mitigated by using NGS in combination with DECS analysis.

In this study, we used a combination of NGS and DECS analysis in an attempt to improve the sensitivity and rapidity of detection of virus-like sequences in *Blueberry shoestring virus* (BSSV)-infected blueberry plants. BSSV has been reported to cause extensive damage to blueberry plants in the U.S.A. and Canada, and has caused huge economic losses through decreased yields [16,17,18,19]. BSSV is included in the quarantine pest list released by the Plant Protection Station of the Japanese government. Moreover, the possible spread of this virus to other countries is being monitored. BSSV is taxonomically classified in the genus *Sobemovirus*. At present, its nucleotide sequence remains to be determined. Hence, we selected this virus to evaluate the combination of DECS and NGS for the detection of a virus whose sequence of which is unknown.

## 2. Materials and Methods

### 2.1. Preparation of Infected BSSV Plants

Four BSSV infected scions were provided by Annemick Schilder, Michigan State University, USA, under a special permit (permit number: 22Y307) issued by the Ministry of Agriculture, Forestry and Fisheries, Japan. BSSV infection of stems and leaves of these scions was confirmed by double antibody sandwich enzyme-linked immunosorbant assay (DAS-ELISA) using a kit specific for BSSV (Agdia Inc., Elkhart, IN, USA). Each of the four scions were grafted onto four individual healthy blueberry seedlings as rootstocks, labeled as No. 1 to No. 4, and cultivated under greenhouse conditions. After confirming that the disease symptoms were present in all BSSV samples, tissues were collected for subsequent experiments.

### 2.2. Library Preparation for NGS

Newly emerged branches weighing about four grams were collected from the blueberry seedlings grafted with BSSV-infected scions, and total nucleic acids were extracted as previously described [20]. Next, 1 mL ISOGEN (Nippon Gene, Tokyo, Japan) was added to total the nucleic acid to extract total RNA according to the manufacturer’s instructions. The obtained total RNA was further purified using an RNeasy Plant Minikit (Qiagen, Hilden, Germany) following the manufacturer’s instructions and subjected to dsRNA extraction following a method previously described [21]. A detailed description of the methods for isolating total nucleic acids from blueberry and for extracting dsRNA are provided in Supplemental Procedure 1.

Using TransPlex Whole Transcriptome Amplification (WTA) Kit (Sigma-Aldrich, St. Louis, MO, USA), extracted dsRNA was reverse-transcribed into cDNA with the supplied random primer following the manufacturer’s protocol, and the cDNA was then amplified. Amplification was carried out using ExTaq DNA polymerase (Takara Bio, Shiga, Japan) and 20 cycles of PCR. Next, the 50 μL WTA products were reacted with 30 μL AMPure XP (Beckman Coulter, Brea, CA, USA), and rinsed twice with 1 mL of 70% ethanol. Thereafter, the products were eluted with 30 μL RNase-free water. In total, 100 ng WTA products were used to construct the NGS library using the Ion Xpress Plus Fragment Library Kit and sequenced on the Ion PGM system (Thermo Fisher Scientific, Waltham, MA, USA). WTA products including various sizes of DNA fragments were digested to 200 bp using Ion shear Plus Reagents Kit (Thermo Fisher Scientific), according to the manufacturer’s instructions.

### 2.3. NGS Sequencing and Data Analysis

Analysis of sequence data was performed using Genomics Workbench ver.7.0 (CLC Bio, Tokyo, Japan). First, the mapping analysis was performed using the complete genomes of all viruses and viroids registered in the NCBI database (Complete RefSeq release of viral and viroid sequences; http://www.ncbi.nlm.nih.gov/genome/viruses/) comprising 5660 sequence data. Second, *de novo* assembly analysis was performed to obtain contigs. We performed BLAST search analyses for the obtained contigs to identify their similarities to known virus sequences. A phylogenetic analysis was conducted by the neighbor-joining method using Mega 6.0 software with a bootstrap resampling analysis (1000 replications), and the sobemovirus-like nucleotide sequences were compared with those of other known species of the genus *Sobemovirus*, using the information available in the NCBI database. The known sobemoviruses include *Cocksfoot mottle virus* (CoMV, NC_002618.2), *Rice yellow mottle virus*-CI (RYMV, NC_001575.2), *Imperata yellow mottle virus* (IYMoV, AM990928.1), *Velvet tobacco mottle virus* (VTMV, NC_014509.2), *Lucerne transient streak virus* (LTSV, NC_001696.2), *Subterranean clover mottle virus* (SCMoV, NC_004346.1), *Southern bean mosaic virus* (SBMV, NC_004060.2), *Sesbania mosaic virus* (SeMV, NC_002568.2), *Sowbane mosaic virus* (SoMV, AM940437), *Southern cowpea mosaic virus* (SCPMV, NC_001625.2), *Ryegrass mottle virus* (RGMoV, NC_003747.2), and *Turnip rosette virus* (TRoV, NC_004553.3). In addition, Cocksfoot mild mosaic virus (CMMV, NC_011108.1), a tentative species of sobemovirus, was included with *Potato leaf roll virus* (PLRV, AF453394.1) as an outgroup, which is a member of the genus *Polerovirus*.

### 2.4. RT-PCR

We designed new primers for RT-PCR analysis based on the sequence data obtained in the NGS analysis; the new primers shared similarity with genomic sequences of *Sobemoviruses* (Table S1). Total RNA for RT-PCR analysis was extracted using an RNeasy Plant Mini kit (Qiagen) as per the manufacturer’s instructions. Reverse-transcription and PCR reaction were performed using Transcript M-MLV (Wako, Osaka, Japan) and LATaq (Takara Bio), respectively, following the manufacturers’ instructions. The reverse transcription involved incubation at 25 °C for 10 min, followed by 37 °C for 45 min. PCR was performed with the following program on Verti200 96-well Thermal cycler (Thermo Fisher Scientific): 98 °C for 5 min, followed by 40 cycles of 98 °C for 1 min, 50 °C for 0.5 min, and 68 °C for 2 min; an additional elongation step at 72 °C for 7 min followed by storage at 4 °C. PCR products were separated on a 1.5% agarose gel, stained with 0.5 μg/mL ethidium bromide, and visualized under UV light.

TaqMan real-time RT-PCR was developed for rapid detection of BSSV with high sensitivity. Primers and probe for TaqMan method were designed using the Primer Express^®^ Software v3.0.1 (Thermo Fisher Scientific) (Table S1). The analyzed samples were obtained from new branches of the four BSSV-grafted blueberry seedlings, the four original BSSV infected scions, and 20 healthy seedlings. Total nucleic acid was extracted from 0.1 g of each plant sample using the SDS-potassium-acetate method as previously described [22], and then dissolved in 100 μL RNase-free water. TaqMan real-time RT-PCR analysis was performed using a TaqMan RNA-to-Ct 1-Step Kit (Thermo Fisher Scientific) as per the manufacturer’s instructions. For each sample, 2 μL total nucleic acid was added to the reaction mixture contained in 96-well plates for the Step One Plus real-time PCR system (Thermo Fisher Scientific). The RNA positive control was transcribed using the MEGAscript RNAi Kit (Thermo Fisher Scientific) from a cDNA fragment of BSSV amplified by PCR with the primers BSSV-PosiCon-F and BSSV-PosiCon-R. The concentration of the positive control RNA was adjusted to 0.1 ng/μL, and 2 μL was added to the reaction mixture.

### 2.5. Generation of a Recombinant BSSV Coat Protein and Its Detection by Immunoblotting

The putative coat protein (CP) gene of BSSV was amplified from cDNAs of BSSV-infected blueberry plants by PCR using pET-BSSV-CP-F and pET-BSSV-CP-R primers (Table S1). The *Pepper mild mottle virus* (PMMoV) CP gene was amplified from pBI-PIW-CP [23], using pET-PIW-CP-F and pET-PIW-CP-R primers (Table S1). PCR was performed using KOD-plus polymerase (TOYOBO, Osaka, Japan) according to the manufacturer’s protocol. The PCR products were introduced into a penta-His tagged protein expression vector pET28a (Merck Millipore, Darmstadt, Germany) using the following restriction enzymes: NheI and XhoI for BSSV-CP, and NdeI and XhoI for PIW-CP. Recombinant proteins were expressed by culturing *Escherichia coli* strain Rosetta (DE3) (Merck Millipore, Billerica, MA, USA) harboring each expression vector for 3 h at 30 °C in the presence of 1 mM isopropyl-β-d(–)-thiogalactopyranoside. His-tagged proteins were purified using TALON^®^ Metal Affinity Resin (Takara Bio) according to the manufacturer’s protocol. His-tagged CPs were separated by SDS-polyacrylamide gel electrophoresis (PAGE) with NuPAGE Novex 12% Bis-tris Gels (Thermo Fisher Scientific). Both purified recombinant CPs were detected by immunoblotting using a previously described method [24] with a modification that involved the use of penta-His HRP conjugate kit (Qiagen, Venlo, The Netherlands). Anti-BSSV rabbit polyclonal antibody (Agdia Inc., Elkhart, IN, USA) and goat anti-rabbit HRP conjugate (Bio-Rad, Hercules, CA, USA) were used for the specific detection of BSSV-CP.

## 3. Results

### 3.1. Detection of the BSSV Genomic Sequence in NGS Data Analysis

**Figure 1:**
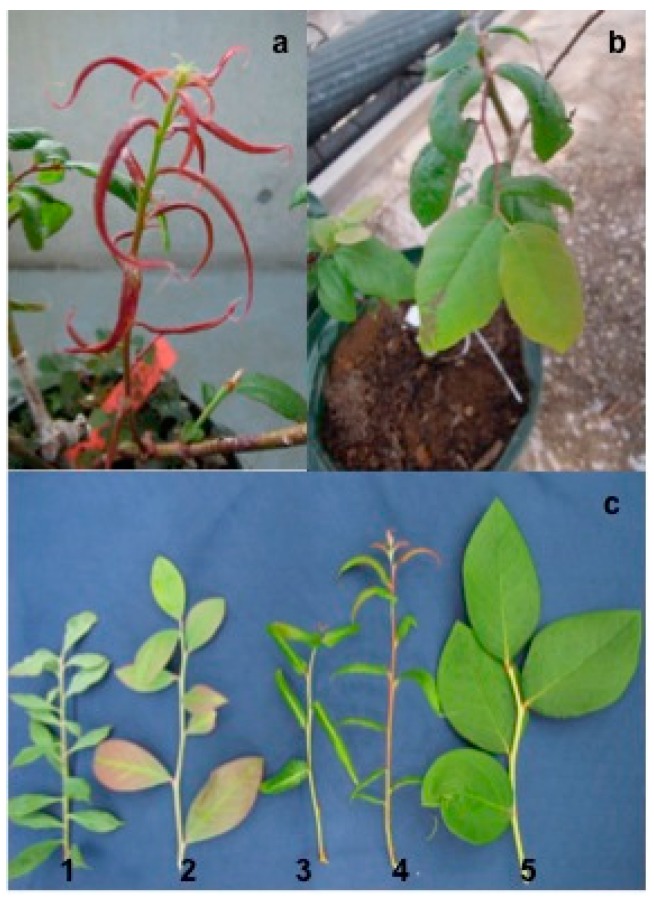
*Blueberry shoestring virus* (BSSV)-like symptoms in grafted blueberry seedlings three years after graft inoculations were performed. (**a**) The blueberry leaves are red and are narrow; (**b**) The main vein of the blueberry leaf has changed to red and there is deformation of the leaf; (**c**) 1–4: Appearance of blueberry seedlings grafted with different BSSV-infected scions. 5: Healthy blueberry leaf.

BSSV infection in the stems and leaves of the infected blueberry scions was examined using ELISA. The stem samples tested positive for BSSV infection whereas the leaf samples tested negative. Although the grafted blueberry seedlings showed no symptoms of BSSV for one year, the leaves were red and narrow, or had turned red in the main vein (Figure 1) after three years. In addition, ELISA confirmed that newly emerged branches of four grafted seedlings were positive for BSSV; no viral sequences were detected in non-grafted seedlings. The symptoms exhibited by newly emerged young branches of seedlings grafted with the No. 2 scion were milder than those grafted with other scions; therefore, we selected newly emerged branches from No. 1 seedling to extract dsRNAs and construct the library for subsequent NGS analysis.

Sequencing with the Ion-PGM system was performed using the library constructed from the obtained dsRNA and 353,824 sequence reads was obtained. Several reads showed a high homology with the genus *Sobemovirus*, as revealed by the mapping tools of Genomics Workbench ver.7 (Table 1). In summary, 117 reads were mapped to the region between nucleotide number (nt No.) 2320 and 2658 of SBMV, a single read was mapped between nt No. 2,977 and 3,165 of RYMV, 94 reads mapped between nt No. 3058 and 3315 of IYMoV, and 12 reads mapped between nt No. 2498 and 2725 of SCPMV. In addition, 4864 reads covered 17,796 nts of the 17,798 nts genome of Blueberry virus A (BVA), a member of the family *Closteroviridae* [25,26], with an average coverage of 36.60. As expected, the NSG enabled us to obtain many more virus-like sequences than the preliminary DECS analysis performed beforehand, in which only three out of the 32 clones sequenced showed similarities with *Sobemovirus*, and no clone with similarities to other viruses was detected [20].

**Figure 2:**
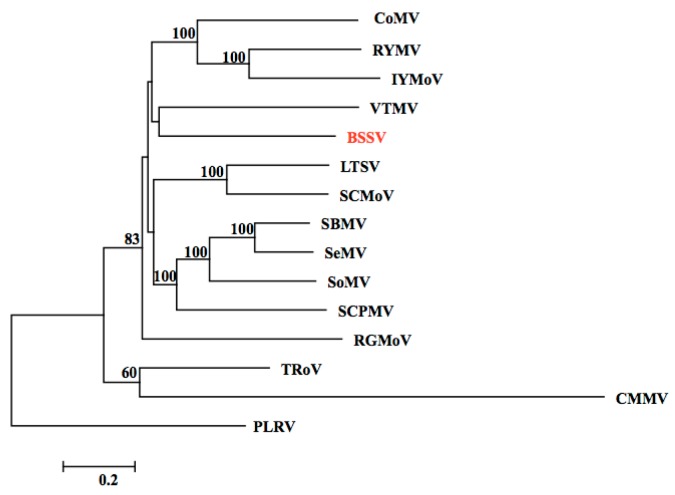
A phylogenetic dendrogram based upon alignments of the full-length genome sequence of *Blueberry shoestring virus* (BSSV) and the known viruses of the genus *Sobemovirus*. The viruses are: *Cocksfoot mottle virus* (CoMV, NC_002618.2), *Rice yellow mottle virus*-CI (RYMV, NC_001575.2), *Imperata yellow mottle virus* (IYMoV, AM990928.1), *Velvet tobacco mottle virus* (VTMV, NC_014509.2), *Blueberry shoestring virus* (BSSV, LC081344), *Lucerne transient streak virus* (LTSV, NC_001696.2), *Subterranean clover mottle virus* (SCMoV, NC_004346.1), *Southern bean mosaic virus* (SBMV, NC_004060.2), *Sesbania mosaic virus* (SeMV, NC_002568.2), *Sowbane mosaic virus* (SoMV, AM940437), *Southern cowpea mosaic virus* (SCPMV, NC_001625.2), *Ryegrass mottle virus* (RGMoV, NC_003747.2), *Turnip rosette virus* (TRoV, NC_004553.3), *Cocksfoot mild mosaic virus* (CMMV, NC_011108.1), and *Potato leaf roll virus* (PLRV, AF453394.1).

Next, using the *de novo* assembly tools of Genomics Workbench ver.7.0, we obtained a contig of 4020 nt that consisted of 9834 reads and shared similarity with members of the genus *Sobemovirus*. All of the reads that matched to sobemoviruses in mapping analysis were present in the contig, which showed 359-fold sequencing coverage. We therefore regarded this contig to be a successful assembly from NGS reads of the genome of BSSV (accession No. LC081344). The putative BSSV genomic sequence shares up to 52% identity with other sobemoviruses (Table S2); 75% identity in full-length nucleotide sequence is a taxonomical border to classify into same species in sobemoviruses. And the putative BSSV genome was most similar to VTMV in a phylogenetic dendrogram of the genus *Sobemovirus* (Figure 2). Comparative analysis with the genomic structures of the other sobemoviruses indicated that the candidate BSSV genome had a structure identical to that of other *Sobemovirus* species with four putative ORFs (ORF1, ORF2a, ORF2b, and ORF3) predicted. ORF1 (nt No. 85-492), ORF2a (nt No. 462-2204), ORF2b (nt No. 1796-3400), and ORF3 (nt No. 3186-4001) were predicted to encode a 15.4 kDa movement protein, a 65.55 kDa polyprotein protein (Protease-VPg), a 62.22 kDa RNA-dependent RNA polymerase protein, and a 27.85 kDa coat protein, respectively. Each sequence of ORFs was compared with those of other sobemoviruses (Table S2); there was no particular tendency for sequence similarity with other sobemovirus in four ORFs. The predicted 5′ and 3′ untranslated regions in the putative BSSV genome are 84 and 19 nucleotides, respectively.

### 3.2. Identification of the BSSV Genome

**Figure 3:**
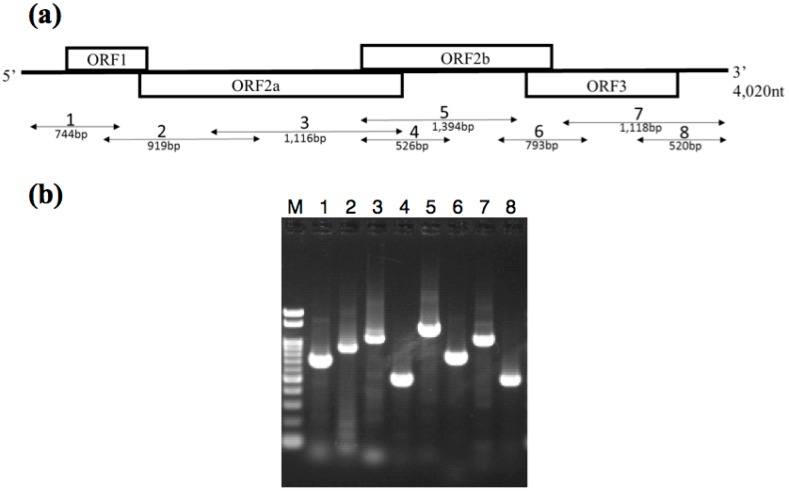
Reverse transcription-polymerase chain reaction (RT-PCR) products obtained from RNA extracted from BSSV-infected blueberry seedlings (**a**) Schematic representation of the genome structure of BSSV and the amplified region of the BSSV genome sequence after PCR using each primer pair. ORF1, movement protein; ORF2a, polyprotein (Protease-VPg); ORF2b, RNA-dependent RNA polymerase; CP, coat protein; (**b**) cDNA fragments of BSSV obtained by RT-PCR using each primer pair. Lane 1, Primers of BSSV-F1/BSSV-R2; Lane 2, BSSV-F2/BSSV-R3; Lane 3, BSSV-F3/BSSV-R4; Lane 4, BSSV-F4/BSSV-R4; Lane 5, BSSV-F4/BSSV-R5; Lane 6, BSSV-F5/BSSV-R5; Lane 7, BSSV-F6/BSSV-R6; Lane 8, BSSV-F7/BSSV-R6; M, 100-bp ladder marker.

The entire region of the candidate BSSV genome sequence was amplified from the cDNA obtained from the BSSV-infected plant using new primer sets that were based on sequences obtained in the NGS analysis (Figure 3). In addition, TaqMan real-time RT-PCR was used to detect BSSV genome candidates. Using this method, the candidate BSSV sequences were successfully detected in all of the samples showing BSSV symptoms, but not in healthy blueberry seedlings (Table 2), and non-specific reactions did not occur. These results strongly suggest that the candidate BSSV genome sequence can be detected specifically in BSSV-infected plants.

**Figure 4:**
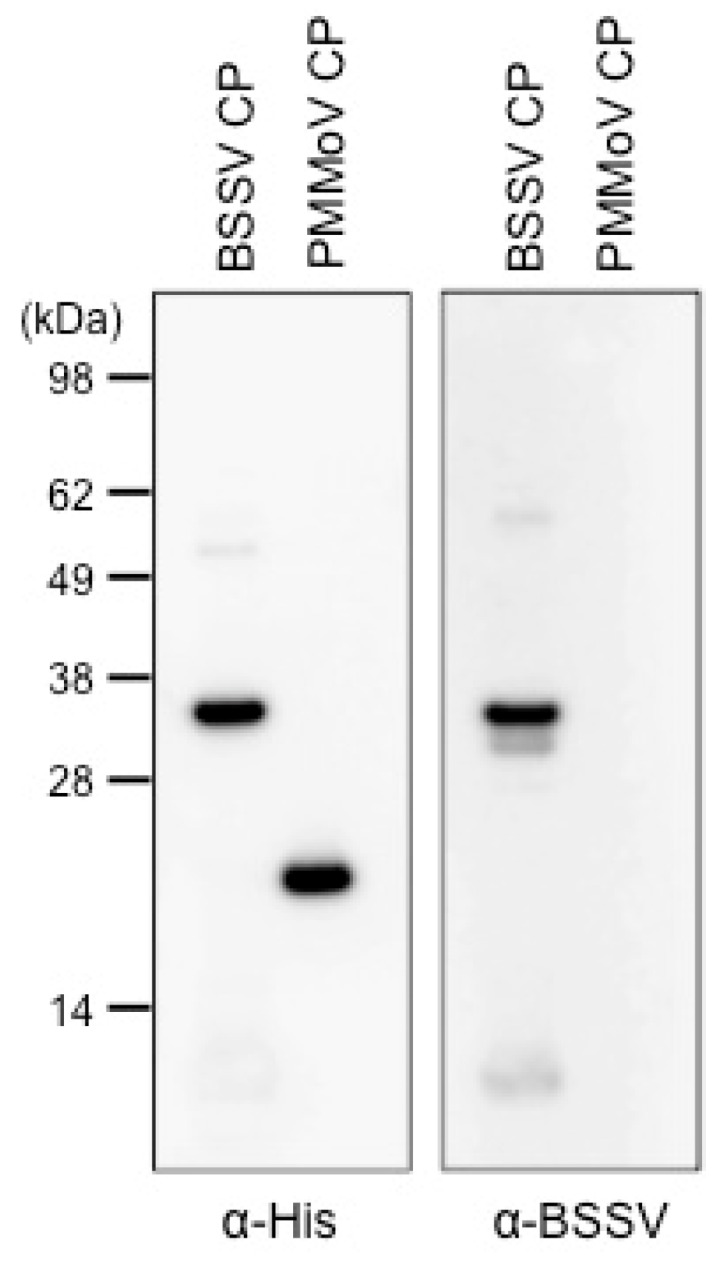
Detection of a recombinant putative coat protein of BSSV using an immunoblotting assay. The left panel shows recombinant proteins detected with an anti-histidine antibody. The right panel shows recombinant proteins detected with an anti-BSSV-antibody.

To further confirm that the candidate sequence equates to the BSSV genome, we tested whether the putative CP was recognized with a commercial BSSV antibody. A recombinant CP expressed from the putative BSSV CP gene was successfully detected by an immunoblot assay using the BSSV antibody (Figure 4). A recombinant PMMoV CP, which was expressed using the same vector and had the same His-tag as the recombinant BSSV CP, did not react with the anti-BSSV antibody, although both recombinant CPs reacted with anti-His-tag antibody (Figure 4). The results strongly supported our conclusion that the sequence we obtained from the NGS analysis was the BSSV genome.

## 4. Discussion

In this study, we have successfully and efficiently detected the BSSV sequence using DECS analysis combined with NGS. The procedure for screening NGS data for virus-like sequences includes the filtering out of plant genome sequences, *de novo* assembly of virus-like sequence candidates, and a BLAST search of the virus sequence database [27,28,29]. Here, we found that plant viruses could readily be detected by simply mapping sequence reads to the reference genomes of virus and viroids registered in the NCBI database using Genomics Workbench. This finding indicates that if a virus of interest belongs to a known genus, then it can readily be detected by a simple mapping procedure. Furthermore, we obtained almost the complete genomic sequence of BVA, indicating that a simple mapping procedure can reveal the complete structure of a new strain or an isolate of a known virus species. We also obtained the almost complete sequence of the BSSV genome through *de novo* assembly of NGS data, confirming the utility of NGS in characterization of virus genomes. The original DECS protocol includes a cloning process, which is time consuming and laborious and requires facilities for recombinant DNA experiments. Using NGS technology, the cloning process before sequencing can be eliminated in the DECS analysis. We would like to propose a name for the “clone-free” DECS analysis combined with NGS protocol as “DECS-C”. Recently, NGS has become more convenient than before, because its cost is decreasing every year. We believe that DECS-C can be a powerful and convenient method to search for plant RNA viruses.

For diagnostic purposes, only part of the virus genome is required for proving the presence of a virus in the plant tissue. Therefore, we may need fewer reads for diagnosis, which would enable us to mix and analyze multiple different libraries in a single NGS run to reduce operational costs. Nevertheless, we cannot rule out the possibility that other viruses might exist in plant materials of interest in quite low amounts and contribute to disease development. It will be necessary to concentrate viral sequences during NGS library preparation in order to enhance detection sensitivity. Although our present protocol used a recombinant dsRNA-binding protein alone to enrich dsRNA, additional treatments, such as nuclease digestion in the presence of higher concentrations of salt, could improve the quality of dsRNA preparation and the efficiency of virus detection with a smaller number of NGS reads. In addition, the operational cost could also be reduced if the exhaustive amplification step before library preparation is omitted. Our current protocol included two amplification steps: first, the amplification of the dsRNA-derived cDNA using a WTA kit; and second, emulsion PCR or bridge amplification depending on the type of NGS. Different manufacturers have released library construction kits for small amounts of RNA for NGS analysis, and these likely make it possible to omit the first amplification step. Collectively, our next challenges should include the improvement of dsRNA quality, the simplification of the NGS library construction, and the reevaluation of the number of sequence reads required to detect plant viruses. We anticipate that DECS-C will evolve through these efforts to become a more powerful, convenient, and cost-effective method, which could be used not only for research purposes but also for routine diagnosis of plant virus diseases [30].

The protocol developed in this study, involves a BSSV detection method with quantitative RT-PCR based on the sequence of only one BSSV strain. It would be preferable to design primers based on highly conserved regions of the genomic sequences of different strains for a more reliable diagnostic method, because RNA viruses are known to evolve rapidly [31]. Therefore, we recommend sequencing samples from different plants showing similar symptoms either in the same field or preferably from different fields. The almost complete BSSV genome sequence obtained here was derived from an assembled sequence from the NGS data; it is possible that this sequence contains errors that have been introduced during the sequencing. Conventionally, the full-length genome sequences of viruses are obtained after purification of the virus particles and genomic sequencing, including both 5′- and 3′-RACE analyses. However, a higher redundancy of sequence reads would mask such artificial errors. It should be possible to minimize errors in the early steps of library construction, which contribute to errors in the final sequence, by the improving the dsRNA yield and by eliminating the initial amplification step of DECS-C. Furthermore, increased biological replicates would guarantee that the sequence data correctly reflect the genome sequences of the virus population in the field. Therefore, the aforementioned improvement of DECS-C will increase its applicability for a variety of purposes.

## Figures and Tables

**Table 1:** Virus-like sequence reads mapped with the species of the genus *Sobemovirus* in next-generation sequencing (NGS) sequence date of *Blueberry shoestring virus* (BSSV) grafted blueberry seedling.

Virus Name	Reference Length (nt)	Mapped Region (nt)	Total Mapped Reads	Average of Coverage	Accession No
*Southern bean mosaic virus*	4132	339	117	4.12	NC_004060.2
*Sesbania mosaic virus*	4148	186	1	0.05	NC_002568.2
*Southern cowpea mosaic virus*	4193	228	12	0.3	NC_001625.2
*Velvet tobacco mottle virus*	4247	201	2	0.09	NC_014509.2
*Rice yellow mottle virus*	4449	189	25	0.77	NC_001575.2
*Imperata yellow mottle virus*	4547	258	94	3.60	NC_011536.1

**Table 2:** Screening of BSSV grafted blueberry seedlings, BSSV sent blueberry scions and healthy blueberry nurseries using TaqMan real-time RT-PCR to assay for BSSV.

Sample Name	Sample No.	Value of Ct ^a^
BSSV-infected scion-grafted seedling	1	13.184
	2	21.951
	3	13.485
	4	12.619
BSSV-infected original scion	1	18.555
	2	13.635
	3	18.922
	4	16.898
Healthy seedling	1~20	N.D. ^b^
NTC ^c^	-	N.D.

^a^ average of Ct value of two replicates, ^b^ non detection; ^c^ negative control.

## Supplementary Materials

The following are available online at http://www.mdpi.com/1999-4915/8/3/70, Table S1: Primer lists. Table S2: Sequence identities and similarities of the putative BSSV genome to those of other sobemoviruses. Supplementary Procedure 1: Isolation of dsRNA using dsRNA-binding protein.
