# Supplementary material for: Combined DECS Analysis and Next-Generation Sequencing Enable Efficient Detection of Novel Plant RNA Viruses
Source: Viruses. 2016 Mar 7;8(3):70. doi: 10.3390/v8030070 (PMC4810260; doi:10.3390/v8030070)
Supplement: Supplementary file 1 [file viruses-08-00070-s001.pdf]

viruses

MDPI

Supplementary Materials: Combined DECS Analysis and Next-Generation Sequencing Enable Efficient Detection of Novel Plant RNA Viruses

Hironobu Yanagisawa [1,2], Reiko Tomita [3], Koji Katsu [2], Takuya Uehara [2], Go Atsumi [3,4], Chika Tateda [3], Kappei Kobayashi [5] and Ken-Taro Sekine [3,*]

Supplementary Procedure 1: Isolation of dsRNA Using dsRNA-Binding Protein

1. RNA Extraction and Purification

Total nucleic acids were extracted from a blueberry by the improved K-SDS method [22]. Briefly about 4 g scions were collected from blueberry seedlings, and crushed using a Multi-beads shocker (YASUI KIKAI CO., Osaka, Japan) for 30 s at 2,000 rpm in liquid nitrogen. Then, 8 mL of extraction buffer solution (100 mM Tris-HCl, pH 8.0, 500 mM NaCl, 1% 2-mercaptoethanol, 50 mM EDTA, pH 8.0, 3.3% Polyvinylpyrrolidone M.W. 4000) was added and centrifuged at 2,500 × g, 4 °C for 10 min. Next, 2.5 mL of homogenate was put into a 15-mL microtube and 0.2 mL 25% SDS was added and stirred well. Thereafter, it was incubated at 65 °C for 10 min, and 1.0 mL 5 M potassium acetate was added and incubated on ice for 20 min. It was centrifuged at 13,000 × g, 4 °C for 10 min, and 0.9 mL of the supernatant was added to each of the two new 1.5-mL microtubes. After adding 0.54 mL of 2-propanol to the supernatant, the solution was mixed well and incubated on ice for 10 min. It was centrifuged at 20,000 × g, 4 °C for 15 min and the supernatant was removed. Following the addition of 1 mL of 70% ethanol, the mixture was centrifuged at 20,000 × g, 4 °C for 5 min and the supernatant was removed. The total nucleic acid was dried under reduced pressure for about 3 min and then dissolved in 100 μL RNase-free water. Next, 1 mL ISOGEN (Nippon Gene) was added to total the nucleic acid to extract total RNA according to the manufacturer's instructions. The obtained total RNA was further purified using an RNeasy Plant Minikit (Qiagen) following the manufacturer's instructions and subjected to dsRNA extraction.

2. Capture of Viral dsRNA

DsRNAs were captured using the GST-DRB4* protein as previously described [5]. The nucleic acid samples were mixed with 18 μg GST-DRB4* in 0.8 mL dRBB (0.1 M Tris–HCl pH 7.0, 0.1 M NaCl, 10 mM $MgCl_2$), added with glutathione-Sepharose (GE Health Care, Chalfont St. Giles, UK; suspended in dRBB containing 0.1% BSA) and incubated for 30 min. After the Sepharose beads were wash 3 times with dRBB containing 0.1% Tween 20 and twice with dRBB. Then nucleic acids bound to the beads were eluted with 100 μL TE buffer containing 0.5% SDS and 5 mM EDTA. The eluted nucleic acids was extracted with phenol/chloroform and precipitated with ethanol. The pellet was dissolved in 20 μL TE buffer, denatured by boiling for 5 min. and quickly chilled on ice water.

Table S1. Primer lists.

Primer Name	Sequence (5′-3′)	Sequence Position	Amplification Size (bp)
BSSV-F1	TTTGGTGTACCAACTGCTTAATTGC	11–35	744
BSSV-R2	ATTGATCACCACGCTGCATG	735–754	
BSSV-F2	CATCACCAATCAAGCCTC	518–535	919
BSSV-R3	TGTCGTCGTAGTCAATCTCA	1417–1436	
BSSV-F3	CATCACCAATCAAGCCTC	1116–1133	1116
BSSV-R4	GTGTCTCGGAGCAACTC	2215–2231	
BSSV-F4	CCCTCAATCAGATTGGCTC	1706–1724	526
BSSV-R4	GTGTCTCGGAGCAACTC	2215–2231	
BSSV-F4	CCCTCAATCAGATTGGCTC	1706–1724	1394
BSSV-R5	TACAATTCTCGGCCAAG	3083–3099	
BSSV-F5	TGTCAAGCAGGAGCCCCACC	2307–2326	793
BSSV-R5	TACAATTCTCGGCCAAG	3083–3099	
BSSV-F6	GTCCCTTGCAGTGTTGGTC	2911–2929	1118
BSSV-R6	TGTGTTTGTCAGGCACGG	4011–4028	
BSSV-F7	CGATGGCTGCGGCTGCGTG	3509–3527	520
BSSV-R6	TGTGTTTGTCAGGCACGG	4011–4028	
pET-BSSV-CP-F	GAAGCTAGCATGGCGACCCGGCTAAG	3163–3180	816
pET-BSSV-CP-R	CTCGAGTCAACAGGATGAGCAACTGCCC	3958–3978	
pET-PIW-CP-F	CATATGGCTTACACAGTTTCCAGTGCCAATC		
pET-PIW-CP-R	CTCGAGAGGAGTTGTAGCCCAGGTGAGTCC		
BSSV-qF	CCGTTCAGGACTGGGAGTTG	2573–2592	81
BSSV-qR	CATTCGCCGTCTCATCACATA	2633–2653	
BSSV-qP	(FAM)-CCGACGTTGAAATGCGCATATCCCTT-(TAMRA)	2597–2622	
BSSV-PosiCon-F	TAATACGACTCACTATAGGGAGATTCACGAGGTGCG ATTTAAGC	2504–2524	200
BSSV-PosiCon-R	TAATACGACTCACTATAGGGAGACCTGTGTTGGAAG ACGGAGTTC	2682–2703	

Table S2. Sequence identities and similarities of the putative BSSV genome compared with those of other Sobemoviuses.

	Full Length	ORF1	ORF2a	ORF2b	ORF3
CoMV	49 [a]	45 [a]/38 [b]	49 [a]/31 [b]	58 [a]/51 [b]	50 [a]/40 [b]
CMMV	45	46/40	44/28	49/34	47/35
IYSV	49	47/50	45/29	55/52	46/42
LTSV	49	45/20	47/29	56/55	46/27
RGMoV	49	47/40	49/83	55/55	45/36
RYMV	49	46/50	45/33	57/54	45/33
SBMV	52	46/57	48/34	60/57	48/44
SCMoV	52	46/33	49/35	57/53	47/38
SCPMV	50	46/42	50/45	59/57	51/34
SeMV	52	46/42	49/71	59/52	50/35
SoMV	50	46/35	49/31	57/59	46/50
TRoV	49	49/44	45/33	57/52	46/33
VTMV	51	46/43	49/30	57/53	48/36
PLRV	45	45/41	43/37	50/48	45/33

[a]: Identities of nucleic acids, [b]: Max identities of amino acids.
